# B cell depletion after traumatic brain injury in 5xFAD mice: influence of biological sex on immune, neuroimmune, and neurobehavioral outcomes

**DOI:** 10.1002/alz70855_105232

**Published:** 2025-12-24

**Authors:** Jaclyn Iannucci, Reagan Dominy, Saloni Tipnis, M. Karen Newell‐Rogers, Lee A. Shapiro

**Affiliations:** ^1^ Texas A&M Health Science Center, Bryan, TX, USA

## Abstract

**Background:**

Traumatic brain injury (TBI) occurs in over 2 million people each year in the United States and is considered a major risk factor for the development of Alzheimer's disease (AD), and related dementias (ADRDs). Identifying shared pathological mediators that contribute to the increased susceptibility of AD following TBI is critically important. Inflammation is considered to play a major role in post‐traumatic syndromes and AD pathogenesis. Previous studies have identified a role for B cells and CLIP+ B cells in TBI pathogenesis, and in the neurobehavioral and pathological outcomes following a fluid percussion injury (FPI) in 5xFAD mice. Therefore, the current study was designed to test the hypothesis that exacerbation of AD‐like pathogenesis and neurobehavioral decline by FPI is dependent on expansion of B cells that can be therapeutically targeted to improve outcomes. Because both TBI and AD exhibit biological sex differences in prevalence and pathology, male and female mice were investigated.

**Method:**

8‐week‐old male and female 5xFAD mice received a single 1.5‐1.8 atm lateral FPI or sham surgery, followed by B cell antibody treatment at 30 minutes and 24 hours post‐injury to deplete early FPI‐induced B cell expansion. Depression‐associated behavior, including burrowing and the social interaction test, were assessed at 3, 6, and 9 weeks post‐FPI. Additional neurobehavioral testing, including elevated plus maze (EPM), Y‐maze, and pattern separation test (PST), was conducted at 10 weeks post‐FPI. Following the conclusion of behavior, tissue was collected to assess neuropathology and immune outcomes.

**Result:**

The results show significant sex differences in the neurobehavioral response to FPI and B cell depletion. These include burrowing and the social interaction test. B cell depletion also improved anxiety behavior in the EPM in male, but not female mice, while in Y‐maze, improvements after B cell depletion were more pronounced in female mice.

**Conclusion:**

These data highlight the therapeutic potential of targeting B cells in TBI and AD, as well as the importance of investigating sex as a biological variable.

